# Phylogeography of the smooth-coated otter (*Lutrogale perspicillata*): distinct evolutionary lineages and hybridization with the Asian small-clawed otter (*Aonyx cinereus*)

**DOI:** 10.1038/srep41611

**Published:** 2017-01-27

**Authors:** Beatrice Moretti, Omar F. Al-Sheikhly, Monica Guerrini, Meryl Theng, Brij K. Gupta, Mukhtar K. Haba, Waseem A. Khan, Aleem A. Khan, Filippo Barbanera

**Affiliations:** 1 Department of Biology, Zoology-Anthropology Unit, Via A. Volta 4, 56126 Pisa, Italy; 2 Department of Biology, University of Baghdad, Al-Jadriya, 10071 Baghdad, Iraq; 3 TRAFFIC Southeast Asia, Unit 3-2, 1st Floor Jalan SS23/11, Taman SEA, 47400 Petaling Jaya, Selangor, Malaysia; 4 Central Zoo Authority, Ministry of Environment, Forest and Climate Change, New Delhi 110003, India; 5 Department of Wildlife & Ecology, University of Veterinary & Animal Sciences, Lahore, Pakistan; 6 Zoology Department, Ghazi University, Dera Ghazi Khan, Pakistan

## Abstract

We investigated the phylogeography of the smooth-coated otter (*Lutrogale perspicillata*) to determine its spatial genetic structure for aiding an adaptive conservation management of the species. Fifty-eight modern and 11 archival (dated 1882–1970) otters sampled from Iraq to Malaysian Borneo were genotyped (mtDNA Cytochrome-*b*, 10 microsatellite DNA loci). Moreover, 16 *Aonyx cinereus* (Asian small-clawed otter) and seven *Lutra lutra* (Eurasian otter) were sequenced to increase information available for phylogenetic reconstructions. As reported in previous studies, we found that *L. perspicillata, A. cinereus* and *A. capensis* (African clawless otter) grouped in a clade sister to the genus *Lutra*, with *L. perspicillata* and *A. cinereus* being reciprocally monophyletic. Within *L. perspicillata*, we uncovered three Evolutionarily Significant Units and proved that *L. p. maxwelli* is not only endemic to Iraq but also the most recent subspecies. We suggest a revision of the distribution range limits of easternmost *L. perspicillata* subspecies. We show that smooth-coated otters in Singapore are *L. perspicillata* x *A. cinereus* hybrids with *A. cinereus* mtDNA, the first reported case of hybridization in the wild among otters. This result also provides evidence supporting the inclusion of *L. perspicillata* and *A. cinereus* in the genus *Amblonyx*, thus avoiding the paraphyly of the genus *Aonyx*.

The Lutrinae subfamily (Carnivora, Mustelidae) comprises 13 species of otters living on all continents except Antarctica and Australasia^1^. Recently, a molecular study carried out by Koepfli *et al*.^2^ provided valuable insight into the phylogeny of otters, confirming an earlier suggestion that Lutrinae was a monophyletic taxon^3^,^4^,^5^. According to Koepfli *et al*.^2^, adaptive radiation of Lutrinae first appeared *c.* 7.5 Ma in Eurasia and involved three main evolutionary lineages. One included the sea otter (*Enhydra lutris*) and river otters from Eurasia (*Lutra lutra*, Eurasian otter; *Aonyx cinereus*, Asian small-clawed otter; *Lutra sumatrana*, hairy-nosed otter; *Lutrogale perspicillata*, smooth-coated otter) and Africa (*Aonyx capensis*, African clawless otter). Another lineage contained New World river otters (genus *Lontra*: four species) while the third lineage, sister to the previous ones and basal within the Lutrinae, comprised the giant otter (*Pteronura brasiliensis*). Furthermore, *L. lutra*-*L. sumatrana* and *A. cinereus*-*L. perspicillata* turned out to be pairs of sister taxa. On the one hand, the placement of *L. perspicillata* as sister to *A. cinereus* was in agreement with results from earlier studies on karyotype, brain structure and fossils of these species^6^,^7^,^8^,^9^,^10^,^11^; on the other hand, such monophyly made *Aonyx* a paraphyletic genus.

The wide distribution range of the smooth-coated otter encompasses socio-politically unstable and remote areas in Asia. Three subspecies are known: *L. p. maxwelli* (Hayman 1956)^12^ in Iraq, *L. p. sindica* (Pocock 1940)^13^ in Pakistan (mostly in the Sindh), and *L. p. perspicillata* (Geoffroy St. Hilaire 1826)^14^ in India, Nepal, and from the Bay of Bengal across Indochina to southwestern Yunnan, the Malaysian Peninsula, Sumatra, Java and Borneo^15^ (Fig. 1). According to the literature, the colour of the coat is the main morphological feature differentiating these subspecies. *Lutrogale p. maxwelli*, which is referred to as the “black otter” by Marsh Arabs, is the darkest taxon, with dark brown to almost black pelage, iron-grey to whitish throat, and light brown to almost grey lower part of the neck and undersides. *Lutrogale p. sindica* holds the palest fur, likely an adaptation to the arid nature of its habitat, with the general hue of the upper side being tawny or sandy brown instead of darker brown with a rusty tinge. In *L. p. perspicillata*, the fur is dark to blackish brown along the back and on the head, while the underside is light brown to almost grey^16^,^17^,^18^.

Listed as Vulnerable by the IUCN and included in Appendix II of CITES, *L. perspicillata* has globally declined by 30% over the past 30 years^19^, meaning that in some place otters are exceedingly rare (e.g., in Iraq) or locally extinct. Major threats include habitat fragmentation and loss, water pollution, overfishing, illegal trapping, trade and hunting^1^,^20^,^21^,^22^,^23^,^24^,^25^.

We investigated the molecular phylogeography of *L. perspicillata* relying on a large sample size collected across the entire species’ range to determine both spatial genetic structure and diversification of the taxon for its management within an adaptive conservation framework^26^. We employed both mitochondrial and microsatellite (Short Tandem Repeats, STR) DNA markers due to their complementary nature, as analyses based on mtDNA alone could reveal only a small part of the evolutionary history of the species^27^. We used the Cytochrome-*b* gene (Cyt-*b*) marker, as the only complete *L. perspicillata* mtDNA sequence available in GenBank concerned this gene^28^. In order to increase geographical coverage, we combined data from modern DNA with those obtained from smooth-coated otter specimens resident in natural history museum collections (archival DNA).

## Results

### Mitochondrial DNA

Two alignments were created, the first comprising 1,131 bp-long Cyt-*b* sequences, the second 305 bp-long fragments of the same gene with all sequences retrieved from museum specimens. We found 32 (H) and 25 (h) haplotypes for the 1,131 and 305 bp-long sequence alignment, respectively, that conformed to a model of neutral evolution (Tajima’s test, *P* > 0.05: *D* = −0.081 and *D* = −0.767, respectively). Sequences showed G-biased nucleotide composition, high transitions/transversions (Ti/Tv) *ratio* (9.66 and 8.78, respectively), and did not contain any internal stop codon and/or indels. Overall, we did not find any evidence for the occurrence of Numts (mitochondrial sequences of nuclear origin^29^). All samples of *L. perspicillata*, except those from Singapore (*A. cinereus* mtDNA), shared maternal ancestry (Fig. 2 and Supplementary Table S1).

### Mitochondrial DNA: 1,131 bp-long sequence alignment

We did not find any saturation in the phylogenetic signal, as the Index of substitution saturation (Iss) value (0.315) was smaller (*P* < 0.001) than that of the critical Iss (Iss.c = 0.753 and Iss.c = 0.470, in symmetrical and asymmetrical trees, respectively). Bayesian (BI), Maximum Likelihood (ML) and Neighbour-Joining (NJ) reconstructions produced identical topologies (Fig. 2). All *L. perspicillata* haplotypes were included in a clade sister to *A. cinereus* (with all Singapore haplotypes). *Lutrogale perspicillata, A. cinereus* and *A. capensis* grouped in a clade sister to the genus *Lutra*, and the estimated divergence time between *A. cinereus* and *L. perspicillata* was 1.33 ± 0.78 Myr (uncorrected p-distance, 0.61 ± 0.36)^3^. Within *L. perspicillata*, we found three distinct, reciprocally monophyletic and statistically well-supported lineages. The first included *L. p. maxwelli* from Iraq, while the second and third comprised otters from South and Southeast Asia respectively belonging to *L. p. sindica* (Pakistan) and *L. p. perspicillata* (India, Bangladesh) and to *L. p. perspicillata* (Thailand, Cambodia, Vietnam, Malaysia) morphological subspecies. Divergence times (as above) were 63 ± 60 Kyr between South and Southeast Asia, 326 ± 152 Kyr between South Asia and Middle East, and 370 ± 174 Kyr between Southeast Asia and Middle East smooth-coated otters.

The most likely reconstruction obtained with Sdiva (Sdiva value = 2,057.7) included (*L.p.sindica*,(*L.p.maxwelli, L.p.perspicillata*)) as the prevailing topology for the smooth-coated otter clade (consensus tree created by Sdiva: Supplementary Figure S1). It was suggested that South East Asia represented the ancestral area (node 44 = 100% D) for the diversification of *L. perspicillata* as well as for the other otters (*L. lutra, L. sumatrana* and *A. cinereus*) occurring in East Asia. The same result was obtained using the command “estimate a node” (node 44: 100% D). The analysis performed with Mesquite and the Bayesian trees with constrained topology within the *L. perspicillata* clade was not successful. However, we found that (*L.p.sindica*, (*L.p.maxwelli, L.p.perspicillata*)) was the topology for which the difference between two states was the closest to 2 (Supplementary Table S5). If that were the case, then South Asia, the state with the lower negative log-likelihood, would have been referred to as the ancestral range for *L. perspicillata*.

### Mitochondrial DNA: 305 bp-long sequence alignment

Three haplogroups were disclosed in the network (Fig. 3). The first (haplotype diversity, *h*: 0.29 ± 0.20) included Middle East otters only, while the second (*h*: 0.60 ± 0.10) and third (*h*: 0.81 ± 0.04) contained otters from South Asia and Southeast Asia, respectively. In the latter, the positive *R*_2_ value (see Methods) was statistically significant (*R*_2_ = 0.134, *P* = 0.03), a population demographic expansion could not be rejected (Mismatch Distribution, MD test: *r* = 0.048; *P*SSD = 0.23), and the McDonald-Kreitman did not detect any sign of purifying selection (*P* = 0.59 and 0.34 with *A. capensis* and *H. maculicollis* as outgroup, respectively). Analysis of Molecular Variance (Amova) showed that haplogroups significantly diverged from each other (ϕ_st_ = 0.83, *P* < 0.001: Table 1), the very large majority of diversity being partitioned among (83.3%) instead of within (16.7%) haplogroups.

### Microsatellite DNA

The STR panel was powerful in discriminating otters^30^ (Probability of identity considering unrelated or sibling individuals: *P*_ID_ = 6.9 × 10^−12^ and *P*_ID_sib = 1.0 × 10^−4^, respectively; Table 2). No evidence for allele dropout and scoring errors was found, and only 2.5% of the microsatellite locus-population combinations turned out to be null alleles. There was no evidence for Linkage Disequilibrium (LE) after Bonferroni correction (*P* > 0.05, all comparisons: Supplementary Table S2). Within *L. perspicillata*, Iraqi otters held the highest number of unique alleles (*A*_u_ = 9) and monomorphic loci (*L*_**_m_**_ = 5) as well as the lowest value of both allelic richness (*A*_r_ = 2.00) and Index of Nei (*I*_**_n_**_ = 0.32). Overall, *A*_u_/*L*_m_ and *A*_r_/*I*_n_ followed an increasing trend from westwards and eastwards, respectively (Table 3). Significant departure from Hardy-Weinberg Equilibrium (HWE) due to heterozygote deficiency was observed in South Asia, Southeast Asia and *A. cinereus* groups (Table 3), which possibly indicated the occurrence of a Wahlund effect^31^. We found that 64.6% of the STR variability was partitioned within *L. perspicillata* haplogroups and 35.4% among them (*F*_**_st_**_ = 0.35, *P* < 0.001), with *F*_st_ pairwise distance values among haplogroups being all highly significant (Table 1).

Bayesian clustering analysis performed with Structure using *L. perspicillata* otters only (Singapore excluded) identified *K* = 3 as the most likely number of genetic clusters (Fig. 4a). Cluster I and II included otters from Iraq and Pakistan/India (Q, average membership probability: Q_I_ and Q_II_ = 1.00, all individuals), respectively, while cluster III contained those from Southeast Asia (Q_III_ range: 0.96–1.00). One Bangladeshi otter showed admixed ancestry (Q_II_ = 0.64; Q_III_ = 0.36) (Fig. 4a).

A second round of clustering analyses revealed a high degree of genetic admixture in the Singapore otter population (Fig. 4b). One individual was assigned to *L. perspicillata* and two to *A. cinereus*, the remaining 15 otters showing admixed genotypes (Q_I_ range: 0.11–0.88) between the parental species (Supplementary Table S3). Average membership probability of the Singapore population to *L. perspicillata* and *A. cinereus* was Q_I_ = 0.42 and Q_II_ = 0.58, respectively.

## Discussion

### *Lutrogale perspicillata* diversification across Asia

The evolutionary relationships of *L. perspicillata* within the Lutrinae perfectly reflected the corresponding part of the phylogeny obtained by Koepfli *et al*.^28^: *L. perspicillata* was placed with *Aonyx* in one clade and *L. lutra* grouped with *L. sumatrana* in another sister to the previous one (Fig. 2). We confirmed the systematic placement of *L. perspicillata* as sister to *A. cinereus* (estimated divergence time: this study, 1.3 Myr; Koepfli *et al*.^2^, 1.5 Myr), and the well-established phylogenetic relationships between these species were further emphasised by the disclosure of *L. perspicillata* x *A. cinereus* hybrids (see below). This result provided additional evolutionary evidence supporting the proposal of Koepfli *et al*.^28^ to include *L. perspicillata* and *A. cinereus* in the genus *Amblonyx* (Rafinesque 1832)^32^. As discussed by these authors, such choice would avoid *Aonyx* to be paraphyletic, thus reflecting monophyly of smooth-coated and Asian small-clawed otters as well as their divergence from the African *A. capensis*.

We found three distinct, reciprocally monophyletic and statistically well-supported *L. perspicillata* evolutionary lineages. The first included *L. p. maxwelli* from Iraq, while the second and third comprised South (*L. p. sindica* + western *L. p. perspicillata*) and Southeast (eastern *L. p. perspicillata*) Asia populations, respectively. These lineages were perfectly concordant with the haplogroups shown in the network (Fig. 3) and with pairwise ϕ_st_ distance values computed among them (Table 1). The large majority (83.3%) of the mtDNA diversity was partitioned among haplogroups instead of within them. The microsatellite DNA confirmed such remarkable spatial genetic structure. Indeed, both pairwise *F*_st_ distance values (Table 1) and Bayesian clustering of individual multilocus genotypes (Fig. 4a) assessed net separation among Middle East, South and Southeast Asia populations. The partition of genetic variation at the nuclear DNA was highly significant (*F*_st_ = 0.35, *P* < 0.001), although most (64.6%) of the diversity was found within haplogroups instead of (35.4%) among them. When we compared mitochondrial *versus* nuclear DNA, we found that the *ratio* of ϕ_st_ to **F**_st_ (0.83/0.35) was 2.4. The most obvious reason for such discrepancy is that mtDNA has a four-time shorter coalescence time than microsatellites, and a decrease in mtDNA diversity should be faster in bottlenecked/declining populations, as it might be the case in *L. perspicillata*^33^.

Ryder^34^ introduced the concept of Evolutionarily Significant Unit (ESU) for prioritising conservation of units below recognised taxonomic levels. Moritz^35^ stressed reciprocal monophyly and divergence of allele frequency at mitochondrial and nuclear DNA loci, respectively, as the most distinctive attributes of an ESU. In this study, we uncovered three ESUs within *L. perspicillata*: Middle East, South Asia and Southeast Asia (Figs 2 and 3). These operational units should allow conservationists to preserve the evolutionary potential of intraspecific genealogies (“keep options alive”)^36^. ESUs can guide *ex situ* collection curators to pursue separate management of *L. perspicillata* conspecifics belonging to distinct lineages, hence, to identify the most appropriate source populations for reintroduction programs. Regrettably, distinctions among otter populations are sometimes forgotten during reintroductions, although it is known that spatial genealogical structuring may occur because of limited gene flow^21^.

**Figure 1:**
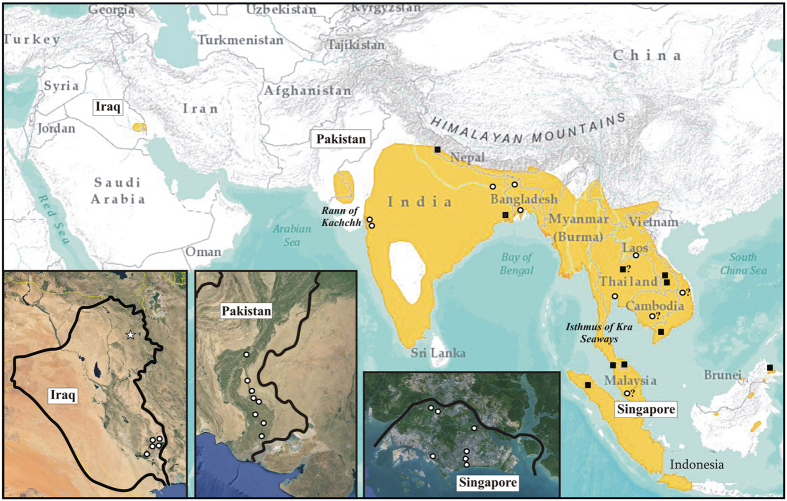
*Lutrogale perspicillata* distribution map including modern (white circles) and archival (black squares) sampling localities (see insets for Iraq, Pakistan and Singapore). In Iraq, the white star indicates the site (TaqTaq, Kurdistan) where the sample of Omer *et al*.^47^ was collected. The positions of the Rann of Kachchh and of the Isthmus of Kra Seaways are reported. Legend:?, unknown locality; PU, Pulau Ubin; PT, Pulau Tekong. See Supplementary Table S1. Geographic ranges were adapted from IUCN (*Lutrogale perspicillata*. The IUCN Red List of Threatened Species. Version 2016-1)^96^. The Figure was modified using CorelDraw! v. 12 (2003) software. Digital images (insets) were obtained from Google Earth v. 7.1.5.1557 (2015 Google Inc.) and modified with CorelDraw! Google Earth map data: [Data SIO, NOAA, U.S. Navy, NGA, GEBCO - Image Landsat] for Iraq and Pakistan digital images, and [Data SIO, NOAA, U.S. Navy, NGA, GEBCO - Image © 2016 Digital Globe - Image © 2016 CNES/Astrium] for Singapore digital image.

**Figure 2:**
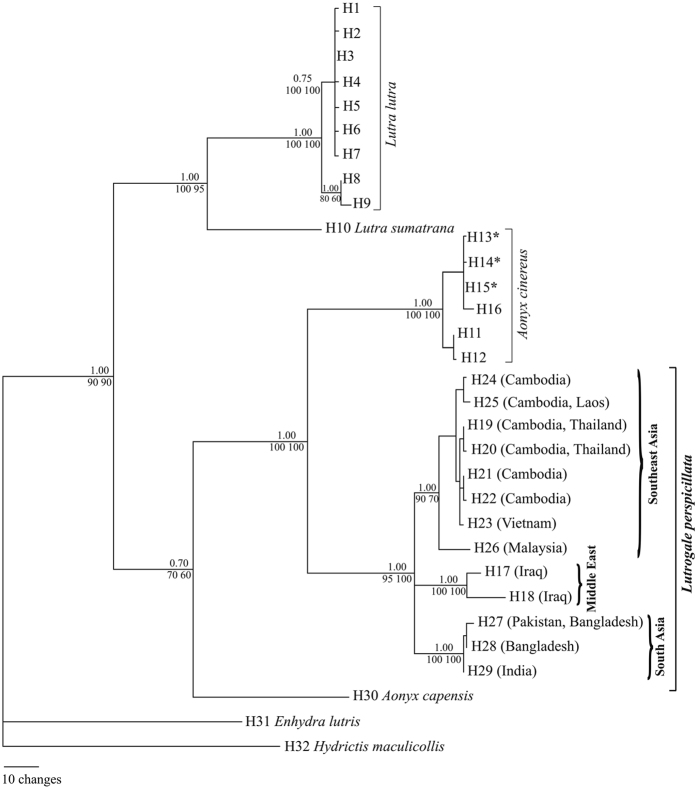
Bayesian (BI) tree computed using modern/GenBank (Supplementary Tables S1 and S4) haplotypes (H, 1,131 bp-long sequence alignment) and *H. maculicollis* as outgroup. Maximum Likelihood (ML) and Neighbour Joining (NJ) methods produced perfectly overlapping reconstructions. Hence, the statistical support was reported at each node as follows: above, posterior probability value computed in the BI analysis; below, bootstrap percentage values computed in the ML (left) and NJ (right) trees. *A. cinereus haplotypes (H13 to H15) from *L. perspicillata* otters sampled in Singapore.

**Figure 3:**
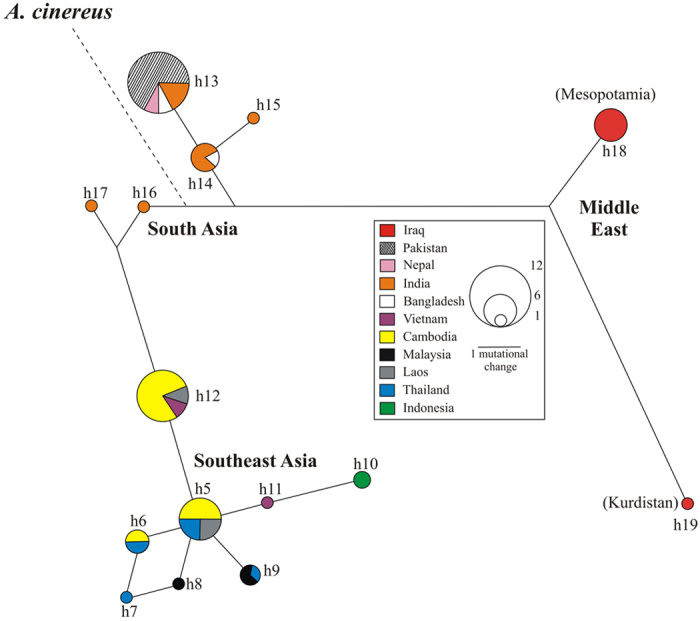
*Lutrogale perspicillata* network computed using haplotypes (h) from the 305 bp-long sequence alignment (modern + archival DNA and GenBank entries). A scale to infer the number of haplotypes for each pie was provided together with a length bar to compute the number of mutational changes. The colour of each country, the number of each haplotype as well as the connection (dashed line) with *A. cinereus* (cf., Fig. 2) are indicated. See Supplementary Tables S1 and S4 for details.

Conventional wisdom suggests that genetic survey results will be more accurate and precise as more samples are employed. We are aware that the biogeographic scenario provided in this study should be considered with caution. Sdiva reconstruction indicated that the most recent ancestor to *L. perspicillata* inhabited Southeast Asia (Supplementary Fig. S1: node 44). This result was in agreement with (i) the East to West decreasing gradient of both mitochondrial (*h*) and nuclear (Table 3) DNA diversity, (ii) the population expansion in Southeast Asia (*R*_2_, MD), and (iii) the comparatively shorter branch length for the Southeast Asia lineage (Fig. 2). Glacial refuges would typically harbour organisms with higher genetic variability than that of derived populations formed by a subset of the original gene pool, and intraspecific diversity should decline away from refuges as consequence of successive founder events during post-glacial colonization^37^,^38^. As already known for many taxonomic groups in Southeast Asia^39^, we found that the haplotypes sampled in the Sundaland (Thai-Malay Peninsula, Sumatra, and Malaysian Borneo: h8 to h10) were private to this region and, as such, distinct from those we found in Indochina (h5 to h7, h11 and h12: all private) (Fig. 3, Table S1). Repeated glacial expansions and retractions might have generated this genetic pattern in *L. perspicillata*, as, for instance, sea level depression was 120 m at the Last Glacial Maximum (20,000 years ago), with a savanna bridge connecting the Thai-Malay Peninsula with Sumatra, Borneo, and Java^39^. Whereas the involvement of the Isthmus of Kra (Fig. 1) can be excluded, as it dissected the Peninsula not later than the 5.5–4.5 Mya^40^, we hypothesised that Southeast Asia might have acted as Pleistocene glacial refuge as well as the source of a westward diversification of *L. perspicillata*. With reference to the latter, however, we found that *A. cinereus*, sister taxon of *L. perspicillata*, was connected to the South instead of Southeast Asia haplogroup (overall star-like structure in the network of Fig. 3). This clearly suggested South Asia as the source for both an eastward and a westward diversification, with *L. p. perspicillata* (to the East) and *L. p. maxwelli* (to the West) as departing subspecies from *L. p. sindica*. Although the analysis carried out with Mesquite did not provide an unequivocal result, we found that (*L.p.sindica*, (*L.p.maxwelli, L.p.perspicillata*)) was the topology for which the difference between two states was the closest to 2 (Supplementary Table S5). If that were the case, then South Asia would have been estimated as the ancestral range for *L. perspicillata*. Despite no definitive proofs are available, we are inclined to consider this scenario (South Asia + eastward and westward diversification) as more reliable than that suggested by Sdiva and the other evidences (South East Asia + westward diversification). Whatever the matter would be, Iraq hosts the most recent subspecies, the divergence time between *L. p. maxwelli* and *L. p. sindica (c*. 330 Kyr) being much longer than that estimated between the latter and *L. p. perspicillata (c*. 60 Kyr).

**Figure 4:**
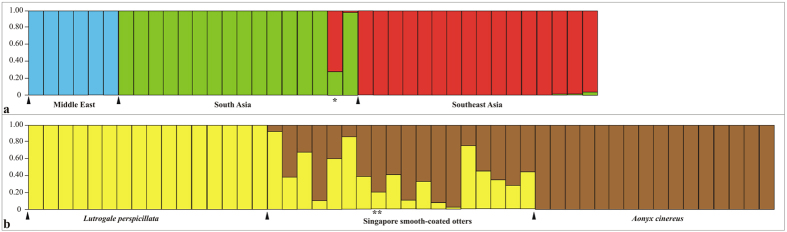
Bayesian analysis of STR multilocus genotypes as computed with Structure. Each individual was represented as a vertical bar partitioned in *K* segments, whose length is proportional to the estimated membership to the *K* clusters. (**A**) *Lutrogale perspicillata* (Singapore excluded), with *K* = 3. Middle East: Iraq; South Asia: Pakistan, India, Nepal and Bangladesh; Southeast Asia: from Thailand to Malaysian Borneo. (**B**) Singapore smooth coated otters compared to either *L. perspicillata* or *A. cinereus* parental control, with *K* = 2 (see text for details). Legend: *individual from southern Bangladesh (Khulna Division): next column to the right refers to the second otter from northern Bangladesh (see Supplementary Table S1); **dead otter found near Kranji Dam, Singapore.

The boundary between *L. perspicillata* easternmost haplogroups and the relationships between Pakistani and Indian populations deserve a comment as well (Figs 3 and 4a). In the first case, Myanmar mountain range and/or rivers (e.g., Brahmaputra) might have restricted the gene flow between Indian sub-continent and Southeast Asia otters, as occurred in many other taxonomic groups^41^,^42^. In the second one, the Rann of Kachchh, a huge seasonally marshy region located between Pakistan (Sindh) and India (Gujarat) (Fig. 1), has likely played a major role. While the Rann connected the fauna of these countries for a long time, the regression of wetlands in the Indian sub-continent caused a marked discontinuity in the distribution of many wet-zone species since the mid-Miocene^43^,^44^. We suggest that the Pakistani smooth-coated otters kept up relic genetic traits of the Indian conspecifics since the gene flow between them was ongoing across the Rann, as occurred with the black francolin (*Francolinus francolinus*, Galliformes)^45^. Therefore, although a more extended sampling coverage as well as ecological data are needed, the distribution range limits of easternmost *L. perspicillata* subspecies might be revised as follows: otters occurring from Pakistan across India North to Nepal and East to Bangladesh should be assigned to *L. p. sindica*, while those inhabiting Indochina and Southeast Asia to *L. p. perspicillata*.

Hayman^12^ described Iraqi smooth-coated otter as a distinct taxon (*L. p. maxwelli*) based on a skin from a dead individual and a young male brought to G.Y. Maxwell by Marsh Arabs. Since then, limited information and no picture of live otters were available. In the 1990 s, the Mesopotamian marshes were drained for political reasons and a catastrophic decline of the local biota has occurred. Despite re-inundation in 2003, otters became exceedingly rare due to hunting, trapping, and habitat loss^24^,^25^,^46^. In this study, we provided consistent DNA evidence for both occurrence and endemicity to Iraq of *L. p. maxwelli*. All genotyped smooth-coated otters were from Mesopotamia; hence, we could not confirm the presence of the species in Kurdistan^47^ (see below). In Iraq, all mtDNA haplotypes and 45% of STR alleles were private and, compared to South and Southeast Asia populations, otters showed the lowest value of haplotype diversity, number of alleles, allelic richness, Nei Index and the highest number of unique alleles (Figs 2, 3 and 4a; Table 3; Supplementary Table S1). On the one hand, this outcome could be due to the small sample size; on the other hand, geographic isolation and related genetic bottlenecks/founder events could have played a major role. Unlike other mammals with uninterrupted distribution across most of southern Asia (e.g., the Indian grey mongoose, *Urva edwardsii*: from Turkey and the Arabian Peninsula East to Bangladesh), *L. perspicillata* is absent between Pakistan and Iraq (no records in central Asia and extinct in Iran^48^). It is likely that such a gap in the species’ distribution range has led to the divergence among *Lutrogale* subspecies (Figs 2, 3 and 4a; Table 1; compare *versus* Fig. 1 in Veron *et al*.^41^).

Omer *et al*.^47^ showed evidence for a smooth-coated otter range extension (*c*. 500 km) towards Kurdistan relying on a single sample (JQ437613: Supplementary Table S4). The latter diverged from the Mesopotamian samples of this study by 8 and 15 nucleotide changes over 305 bp and 1,131 bp, respectively (Figs 2 and 3), a value up to ten times higher than that we disclosed for *L. lutra* from the same areas (zero and < 2 over 305 and 1,131 nucleotide positions, respectively). Moreover, we found only *L. lutra* genetic evidence at the same site surveyed by Omer *et al*.^47^ in Kurdistan (Supplementary Table S1). To conclude, distinct northern and southern *L. p. maxwelli* populations would seem a matter of fact. On the one hand, the incomplete JQ437613 entry (i.e., with nine unresolved nucleotide positions) might suggest some sequencing trouble for the sample in point. On the other hand, mitochondrial sequence diversity is known to be very low in *L. lutra*^28^, hence, our results would be not so surprising. Although further investigations are needed to shed some light on *L. perspicillata* in North Iraq, we feel that *L. p. maxwelli*’s endemicity will be pivotal to draw up a national action plan for the protection of the species^24^.

### Introgressive hybridization with the Asian small-clawed otter

Among animals, 10% of species are involved in hybridization and potential introgression^49^, mustelids not being an exception^50^,^51^. Although mtDNA is more prone to introgression than nuclear DNA^27^, there are many examples of mtDNA capture with (e.g., Barbanera *et al*.^52^) or without (e.g., Bernatchez *et al*.^53^) nuclear introgression. Our study falls in the first case, as wild phenotypic smooth-coated otters sampled in Singapore (Fig. 1, Supplementary Table S1) turned out to be *L. perspicillata* x *A. cinereus* hybrids with *A. cinereus* maternal ancestry (Figs 2 and 4b, Supplementary Table S3). This result represents the first record of introgressive hybridization in a wild otter population worldwide. Nevertheless, the occurrence of tight evolutionary relationships between *L. perspicillata* and *A. cinereus* was known based on molecular phylogenetic, genetic and morphological data (see Introduction). Moreover, to date, the only known hybrid otters were those born in captivity as a result of a crossing between an *L. perspicillata* male with an *A. cinereus* female^54^.

Integration of genetic material from one species (*A. cinereus*) into another (*L. perspicillata*) and morphological resemblance to one parental species only (*L. perspicillata*) suggest repeated backcrossing to the latter. Nonetheless, hybrid otters contained the mtDNA of only one of the parental species, *A. cinereus*. Since the 1960 s, the latter has become gradually rarer than *L. perspicillata* in Singapore and appeared to be more a visitor than a resident species. In this area, at the present time, *A. cinereus* inhabits only off shore islands (Pulau Ubin, Pulau Tekong: Fig. 1)^55^,^56^. We suggest the occurrence of unidirectional hybridization between *A. cinereus* females and *L. perspicillata* males, with either prezygotic or postzygotic mechanisms being potentially responsible for the lack of the *L. perspicillata* maternal line^57^. In the first case, difference in size between smooth-coated (*c*. 11 kg) and Asian small-clawed (*c*. 5 kg) otter males might have worked as sovranormal stimulus for *A. cinereus* females. Indeed, it is most likely to be the female of the smaller species that accepts the male of the larger species than the opposite^58^. According to the “sexual selection hypothesis”, *A. cinereus* females might have initially rejected *L. perspicillata*, but the longer they failed in searching for males of their own species the less discriminating they likely became and, eventually, mated with the male of the common species. In the event of postzygotic mechanisms, *L. perspicillata* (female) x *A. cinereus* (male) crossing could have been unviable or had lower fitness. More likely, even though both parental mtDNAs might have been present initially, one lineage could have gone extinct^57^. *Aonyx cinereus* mtDNA capture could have been due to selective pressure (adaptation) and/or chance (drift), an event that can occur quickly in small and fragmented populations, as it was found, for instance, in the asp viper (*Vipera aspis*^52^). To conclude, further research on sympatric smooth-coated and Asian small-clawed otter populations is needed to establish if hybridization is more widespread than what we know today. The genetic admixture of the Singapore otter population might have implications for its adaptation to the present-day fast changing environment; hence, a genetic survey relying on functional markers (e.g., Major Histocompatibility Complex loci) could be helpful for supporting its long-term conservation.

## Methods

### Biological sampling

We collected 58 *L. perspicillata* samples from Iraq to Malaysian Borneo (Fig. 1). We sampled otters in the wild in Iraq, Pakistan, India, Thailand and Singapore. Although *L. perspicillata* is kept in low numbers in captivity, we also sampled *ex situ* individuals never housed with other otter species and whose origin in the wild was known to collection curators. *Aonyx cinereus* samples (*n* = 16) were obtained mostly from European and Australian zoos, while we collected *L. lutra* samples in Iraq (Kurdistan, *n* = 4) and in Italy (*n* = 3) (Fig. 1 and Supplementary Table S1). However, faeces (“spraints” in otters) and samples collected by veterinary staff members of zoos were also used. Only in Pakistan, samples (blood/hairs) were taken from otters trapped in the wild. Methods were performed in accordance with the relevant guidelines and regulations of the Animal Health and Welfare Regulations (AHWR) of the Bahauddin Zakariya University, and were approved by the Institutional Research Ethical Committee of the same University (permit #D-1/2016). In the light of the type of work done, we did not require approval from the Animal Welfare Body (in Italian, “Organismo preposto al Benessere Animale”) of the University of Pisa.

We borrowed samples from 11 *L. perspicillata* specimens resident in the Field Museum of Natural History of Chicago, in the Smithsonian Institution National Museum of Natural History of Washington D.C., in the Natural History Museum of Denmark (Copenhagen), in the National Museum of Natural History of Paris, and in the Natural History Museum of Vienna. Specimens were collected over a period from 1882 to 1970 (Fig. 1 and Supplementary Table S1). Curators provided a tiny amount (<5 mg) of either dry skin or bone fragments mostly from the skull cavity (e.g., turbinates) or slivers of toe pad.

### DNA extraction

We extracted DNA from modern samples in the Zoology building of the Department of Biology, Pisa. We used DNeasy Blood and Tissue Kit (hair/blood/skin samples) and QIAamp DNA Stool Mini Kit (spraints) following instructions provided by the manufacturer (Qiagen). Reliability of each extraction was checked through negative controls, while DNA concentration and purity were assessed (spraints excluded) with an Eppendorf BioPhotometer (AG Eppendorf). Finally, we extracted DNA from archival samples in a dedicated room free of any mammal DNA in the Anthropology building of the Department of Biology (Pisa) following Forcina *et al*.^59^.

### Mitochondrial DNA

We designed PCR and sequencing Cyt-*b* primers for *L. perspicillata, A. cinereus* and *L. lutra* (modern and archival DNA: Table 4). For the modern samples, we performed PCR reactions as in Guerrini *et al*.^60^ adding 1 μl of 75 μM Bovine Serum Albumin (BSA) (Sigma Aldrich) to all reactions, setting the annealing time to 1 min and including two blank controls. When the amplification was not successful, we obtained the entire Cyt-*b* gene (1,140 bp) by amplifying the purified products of the first PCR via semi-nested PCRs (snPCRs) as reported in Guerrini & Barbanera^61^. In the second PCR, two overlapping fragments (1^st^: 754 bp; 2^nd^: 612 bp) were amplified for each sample in two reaction tubes applying the same thermal profile as in the first PCR. We purified and sequenced all PCR products as in Guerrini *et al*.^60^.

For the archival samples, we amplified two overlapping gene fragments (1^st^: 211 bp, 2^nd^: 199 bp) in two distinct reaction tubes. Each final 307 bp-long sequence corresponded to the Cyt-*b* portion comprised between nucleotide position n. 602 and n. 908 (codon reading frame, 2). We carried out PCR reactions as in Barbanera *et al*.^62^ and we purified/sequenced PCR products as above.

We sequenced the entire Cyt-*b* gene for 56 out of 58 modern *L. perspicillata*, all *A. cinereus (n* = 16) and *L. lutra (n* = 7); for two Indian *L. perspicillata* we obtained the 307 bp-long fragment (Supplementary Table S1). The latter fragment was sequenced for all (*n* = 11) museum samples. Two Cyt-*b* alignments were created using ClustalX 1.81^63^. First (entire gene: 1,140 bp) included 96 sequences (56 + 16 + 7 plus 16 GenBank and one unpublished sequence: Supplementary Table S4). Iraqi JQ437613^47^ contained nine unresolved positions; hence, we used 1,131 nucleotides in the analyses. Then, we created a 307 bp-long sequence alignment including two unpublished, two Indian, all museum and previous sequences (2 + 2 + 11 + 96 = 111: Supplementary Tables S1 and S4). However, we used 305 nucleotides because of the incomplete JQ437613 entry (see above).

We employed Mega 5^64^ to calculate nucleotide composition, to check for internal stop codons/indels, and to compute Ti/Tv *ratio*. We used DnaSp 5.00^65^ to infer haplotypes (H and h for 1,131 bp-long and 305 bp-long sequence alignment, respectively) and to check for neutral evolution of the sequences^66^. GenBank accession codes are reported in Supplementary Tables S1 and S4.

### Mitochondrial DNA: 1,131 bp-long sequence alignment

We evaluated the phylogenetic signal by calculating the Iss (Xia test with 1,000 bootstrap replicates^67^) and plotting the number of Ti and Tv against a TN93 corrected distance^68^ with Dambe 4.2.13^69^. We used Smart Model Selection as implemented in PhyML 3.0^70^ and found that the General Time Reversible (GTR) + G (α = 0.223) was the best evolutionary model fitting to our dataset according to both the Akaike (8,364.0) and Bayesian (8,701.1) Information Criterion. In a Bayesian analysis, however, Metropolis-coupled Monte Carlo Markov Chains (MCMC) integrates over the uncertainty in parameters values. Hence, only the general form of the model was included in the BI performed with MrBayes 3.1.2^71^. Two independent runs of analysis were conducted for 4,000,000 generations with a sample frequency of 100 (four chains, heating = 0.2, random starting tree). Convergence between runs was monitored through the Average Standard Deviation of Split Frequencies (ASDSF) until this value dropped well below 0.01. Stationarity was reached after 400,000 generations (ASDSF = 0.003774) as inferred using Tracer 1.5.0^72^. Hence, 4,000 trees were discarded as burn-in, and the remaining 72,002 trees were used to produce a 50% majority-rule consensus tree. Then, we carried out both ML (GTR + G model, Nearest Neighbour Interchange, automatically generated starting tree) and NJ (parameters estimated with Smart Model Selection) tree reconstructions using Mega and Paup* 4.0b10^73^, respectively. Trees were rooted using AF057125 *H. maculicollis* (spotted-necked otter: Supplementary Table S4) of Koepfli & Wayne^3^, and the statistical support at each node was evaluated by calculating the Posterior Probability value (PP, for BI) and the Bootstrapping Percentage (BP, for ML and NJ, with 10,000 replicates^74^). In the present study, many *L. perspicillata* Cyt-*b* sequences are available for the first time. Hence, we employed the 0.46%/Myr rate (Cyt-*b*: Tv, 3^rd^ position) of Koepfli & Wayne^3^ to date separation between *A. cinereus* and *L. perspicillata* as well as among *L. perspicillata* subspecies, although we are aware that such estimates should be considered as tentative.

We reconstructed historical biogeography of *L. perspicillata* using Sdiva (Statistical Dispersal-Vicariance)^75^ as implemented in Rasp 3.2 (Reconstruct Ancestral State In Phylogenies)^76^. Six regions were set-up (code: A to F): (A) Europe; (B) Middle East; (C) South Asia; (D) Southeast Asia; (E) Africa; (F) northern Pacific coast. When the distribution of each taxon encompassed more than one region, the character state was polymorphic and the maximum number of areas set for the Sdiva output was three. We used the posterior family of topologies obtained from the Bayesian reconstruction with 72,002 trees. Taking into account that the program does not admit politomy, we used either the majority rule consensus tree created by Sdiva with compatible groups with less than 50% support allowed or the command “estimate a node” to evaluate a given node individually. We also carried out three additional Bayesian tree reconstructions as that of Fig. 2 (all parameters) but with constrained topology within the *L. perspicillata* clade: (1) (*L.p.sindica*,(*L.p.maxwelli, L.p.perspicillata*)), (2) (*L.p.maxwelli*,(*L.p.sindica, L.p.perspicillata*)) and (3) (*L.p.perspicillata*,(*L.p.maxwelli, L.p.sindica*)) (each tree: node 41, PP = 1.00). As in Koepfli *et al*.^2^, we investigated these alternative arrangements using the Likelihood Reconstruction method (Markov *k*-state one parameter model) as implemented in Mesquite 3.10^77^. In particular, we employed the likelihood-ratio test to determine the best estimate of the reconstructed character state at node 44 (*L. perspicillata* clade). The regions were set-up with code 0–5 and haplotypes assigned as follows: 0, Europe (H4-H7); 1, Middle East (H1-H3, H17, H18); 2, South Asia (H27-H29); 3, South East Asia (H19-H26, H11-H16, H8-H10); 4, Africa (H30, H32); 5, northern Pacific coast (H31). The likelihood threshold was set at 2.0, namely the ancestral state reconstruction was considered equivocal at the investigated node if log-likelihoods differed by less than 2.0.

### Mitochondrial DNA: 305 bp-long sequence alignment

We constructed a *L. perspicillata* haplotype network using the Median Joining method^78^ with Network 4.6.1.3 (2014–2015 Fluxus Technology, UK). We employed Arlequin 3.5.1^79^ to investigate the partition of diversity among and within haplogroups by Amova using ϕ_st_, analogous to Wright’s^80^ *F*-statistics (10,000 permutations), and to calculate haplotype diversity (*h*) for each haplogroup.

Within *L. perspicillata*, demographic inferences were obtained only for the Southeast Asia haplogroup (see Results), as the others did not include a reliable number of haplotypes for the analyses at issue. Ramirez-Soriano *et al*.^81^ found that the most powerful tests to detect a population demographic change analysing DNA polymorphisms were those based on haplotype frequencies. Among these, *R*_2_ statistics has the greatest power to detect population expansion when the sample size is quite small (<10)^82^. Hence, we estimated the significance of the *R*_2_ statistics through the null distribution of 5,000 coalescence simulations with DnaSp, and we determined the Mismatch Distributions (MD) of mtDNA pairwise distances with Arlequin. As to this latter, the more ragged the shape of the distribution, the closer the population to a stationary model of constant size over a long period (Harpending’s raggedness index, *r*)^83^. The MD test uses the observed parameters of the expansion to perform coalescent simulations and to create new estimates of the same parameters. Departure from a model of sudden expansion was tested by summing the squared differences (SSD) between observed and estimated MD^84^. In the same haplogroup, the McDonald-Kreitman test^85^ was run with DnaSp to investigate the deviation from an equal ratio of non-synonymous to synonymous fixed substitutions using either *A. capensis* or *H. maculicollis* as outgroup (Supplementary Table S4).

### Microsatellite DNA

We genotyped 56 *L. perspicillata* and 16 *A. cinereus* (see below) samples (Supplementary Table S1) at 10 loci originally isolated from the Eurasian otter genome (Table 2). We performed PCRs (12.5 μl) as in Barbanera *et al*.^86^ according to a touch-down thermal profile (Table 2). We added 0.3 μl of 75 μM BSA to all reactions and included two blank controls. We sequenced on both DNA strands at least two alleles per locus to validate each repeated motif (Table 2). We genotyped each locus from two to five times according to the comparative multiple-tubes approach of Frantz *et al*.^87^. We used Gimlet 1.3.3^88^ to confirm each consensus genotype and to evaluate the discriminatory power of the whole set of loci (*P*_ID_ and *P*_ID_sib)^30^. We used Micro-checker 2.2.3^89^ to check for null alleles, allele dropout and to score errors due to stuttering. We used Fstat 2.9.3^90^ to determine the number of alleles (*A*), the number of unique alleles (*A*_u_) and the allelic richness (*A*_r_). We used Genepop 4.2^91^ and Arlequin (i) to calculate the Index of Nei (*I*_n_), expected (*H*_e_) and observed (*H*_o_) heterozygosity, (ii) to infer deviations from HWE and LE, and (iii) to investigate the partition of STR diversity among and within *L. perspicillata* haplogroups (see Results) by Amova using pairwise *F*_st_ distances (10,000 permutations)^80^. We adopted the Bonferroni correction to adjust the significance level of each test^92^.

We used Structure 2.3.4^93^ to estimate the posterior probability of membership of each individual to *K* assumed genetic clusters. First, we investigated the genetic structure of *L. perspicillata* relying on pre-defined haplogroups (see Results) without prior information on the origin of samples and admixture model, with 10^6^ MCMC iterations, a burn-in of 10^5^ iterations, and 10 replicates per each *K*-value (1 to 12). The number of clusters that best fitted to the data was chosen as in Evanno *et al*.^94^, and an identification threshold (Q_i_) to each cluster was set to 0.90^95^.

In a second round of analyses, we inferred genetic identity of phenotypic *L. perspicillata* otters from Singapore (*n* = 18). In the light of their *A. cinereus* mtDNA lineage (see Results), the involvement of the latter was considered the most likely as the counterpart of hypothetical introgressive events. We used *L. perspicillata (n* = 16: Middle East, 2; South Asia, 3; Southeast Asia, 11) and *A. cinereus (n* = 16) individuals as parental controls (Supplementary Table S1). We employed Structure to estimate the posterior probability of each Singapore otter to belong to one parental species or to have fractions (Q_i_) of its genome originating from the two parental species. We enabled the “popflag” option (*K* = 2) targeting *L. perspicillata* and *A. cinereus* as controls and Singapore as the unknown population, namely we requested Structure to only update allele frequencies with the genotypes of known individuals^93^.

## Additional Information

**How to cite this article:** Moretti, B. *et al*. Phylogeography of the smooth-coated otter (*Lutrogale perspicillata*): distinct evolutionary lineages and hybridization with the Asian small-clawed otter (*Aonyx cinereus*). *Sci. Rep.* **7**, 41611; doi: 10.1038/srep41611 (2017).

**Publisher's note:** Springer Nature remains neutral with regard to jurisdictional claims in published maps and institutional affiliations.

## Supplementary Material

Supplementary Information

## Figures and Tables

**Table 1:** ϕ_st_ (mtDNA, above diagonal) and ***F***_**_st_**_**(STR,** below diagonal) pairwise distance values among *L. perspicillata* haplogroups (Fig. 3).

	Middle East	South Asia	Southeast Asia
Middle East	—	0.86	0.83
South Asia	0.50	—	0.78
Southeast Asia	0.45	0.23	—

Middle East: Iraq; South Asia: Pakistan, India, Nepal and Bangladesh; Southeast Asia: from Thailand to Malaysian Borneo. All values, *P* < 0.001.

**Table 2:** Characteristics of investigated STR loci: *T*_a_ (°C), annealing temperature; TD, touch-down PCR; F, forward; R, reverse; size range (bp); *A*, number of alleles; *P*_ID_, probability that two individuals drawn at random share identical genotypes; *P*_ID_sib, probability of identity among siblings; repeated motif.

Locus	Label	*T*_a_ (°C)	Primer sequence (5′–3′)	Size-range (bp)	*A*	*P*_ID_	*P*_ID_sib	Repeated motif
Lut435	HEX	48	F: TGAAGCCCAGCTTGGTACTTC	113–133	11	2.7 × 10^−2^	3.3 × 10^−1^	(CA)_15_
			R: ACAGACAGTATCCAAGGGACCTG					
Lut615	HEX	TD 52–48	F: TGCAAAATTAGGCATTTCATTCC	223–249	10	9.9 × 10^−4^	1.1 × 10^−1^	(GT)_12_
			R: ATTCTCTTTTGCCCTTTGCTTC					
Lut818	FAM	TD 52–48	F: AAGGATGTGAAACAGCATTG	142–184	8	4.5 × 10^−5^	3.9 × 10^−2^	(GATA)_3_
			R: CCATTTTATACACATAAATCGGAT					
Lut457	TET	TD 52–48	F: CAGGTTTATGGCTTTATGGCTTTC	153–175	8	2.1 × 10^−6^	1.4 × 10^−2^	(CA)_9_
			R: CAGGGTTTGATTTCTGGTGAGG					
Lut701	TET	TD 55–52	F: GGAAACTGTTAAAGGAGCTCACC	152–180	10	1.1 × 10^−7^	5.0 × 10^−3^	(CCTT)_2_…(CTAT)_9_
			R: CAGTGTTCATAAGGATGCTCCTAC					
Lut453	FAM	TD 52–48	F: AGTGCTTTGTACTTGGTAATGG	97–131	9	9.8 × 10^−9^	2.0 × 10^−3^	(CA)_9_
			R: AGACTGAAAGCTCTGTGAGGTC					
OT19	FAM	TD 55–52	F: ATAGGTCTCTCAGCACGGTGTCT	203–223	6	1.2 × 10^−9^	8.7 × 10^−4^	(GGAA)_6_..(GAAA)_7_
			R: TTAAATCCACATCTGTGACTCTGCA					
Lut832	TET	TD 52–48	F: TGATACTTTCTACCCAGGTGTC	176–192	5	1.6 × 10^−10^	3.8 × 10^−4^	(GATA)_8_
			R: TCCTTAGCATTATCTTATTTACCAC					
Lut604	FAM	TD 55–52	F: TATGATCCTGGTAGATTAACTTTGTG	97–109	6	2.8 × 10^−11^	1.9 × 10^−4^	(GT)_7_
			R: TTTCAACAATTCATGCTGGAAC					
OT17	HEX	TD 55–52	F: ATCAGGTATGAGGATACATTTACCT	144–162	4	6.9 × 10^−12^	1.0 × 10^−4^	(CTTT)_6_
			R: TGCAACCTACTTCTATATGAATTT					

Loci are sorted according to the increasing order of their *P*_ID_ and *P*_ID_sib single-locus values (i.e., the locus at the top is the most informative one), and a sequentially multi-loci *P*_ID_ (*P*_ID_sib) is reported for each locus. *A, P*_ID_ and *P*_ID_sib values were calculated using the entire *L. perspicillata* modern dataset (Supplementary Table S1). All loci were from Dallas & Piertney^97^ with the exception of OT17 and OT19^98^.

**Table 3:** Genetic variability of STR loci for *L. perspicillata* haplogroups (Fig. 3), Singapore population and *A. cinereus* parental control.

Haplogroup	*n*	*A*	*A*_r_	*A*_u_	*L*_m_	*I*_n_	*H*_O_	*H*_E_	*P*_HWE_	χ^2^ (*df*)
Middle East	6	20	2.00	9	5	0.32	0.67	0.63	0.17	14.1 (10)
South Asia	16	41	3.32	5	1	0.51	0.41	0.59	<0.001	∞ (18)
Southeast Asia	16	51	3.98	2	0	0.63	0.56	0.64	<0.001	70.1 (20)
Singapore	18	44	3.29	1	0	0.51	0.47	0.53	0.032	33.1 (20)
*A. cinereus*	16	60	4.62	20	0	0.67	0.59	0.73	<0.001	49.2 (20)

Legend: *n*, sample size; *A*, number of alleles; *A*_r_, allelic richness; *A*_u_, number of unique alleles; *L*_m_, number of monomorphic loci; *I*_n_, Index of Nei; *H*_O_, observed heterozygosity; *H*_E_, expected heterozygosity; *P*_HWE_, probability value for the Hardy-Weinberg Equilibrium test; χ^**2**^ test with relative degrees of freedom (*df*) (Fischer global test, all loci). Departure from HWE was significant for South Asia, Southeast Asia and *A. cinereus* also after Bonferroni correction (α = 0.05, α′ = 0.05/10 * 5 = 0.001). Middle East: Iraq; South Asia: Pakistan, India, Nepal and Bangladesh; Southeast Asia: from Thailand to Malaysian Borneo.

**Table 4:** Type, name and nucleotide sequence of mtDNA Cyt-*b* primers used in this study; *modified from Irwin *et al*.^99^.

Type	Name	Nucleotide sequence (5′-3′)
Modern DNA: *Lutrogale*/*Aonyx*/*Lutra*		
Entire gene PCR	Lutra_L14724*	TGACTAGTAACATGAAAAATCACGTTG
	Lutra_H15915*	GGGATTCTGCATTTTTGGTTTACAAGAC
Semi-nested PCR and/or sequencing	LutroCb_fw583	GTTCACCTCCTGTTTCTCC
	LutroCb_rev706	AGAAGTAGGGCGCCCAGG
	LutroCb_rev706_Aonyx	AGGAGTAGGGCGCCTAGG
	LutroCb_fw298	CGCGGCCTATACTATGGATC
	LutroCb_rev417	GATTACGGTTGCGCCTCAAAAG
	LutroCb_fw775	GCCAACCCGCTCAGTACACC
	LutroCb_rev906	GTGTGTAGCAGTGGGACGATG
Archival DNA: *Lutrogale*		
PCR and/or sequencing	LutroCb_fw583	GTTCACCTCCTGTTTCTCC
	LutroCb_fw610	GGCTCCAACAACCCCTCCGG
	LutroCb_fw727	GTACTATTCTCCCCAGACCT
	LutroCb_rev746	AGGTCTGGGGAGAATAGTAC
	LutroCb_fw775	GCCAACCCGCTCAGTACACC
	LutroCb_rev794	GGTGTACTGAGCGGGTTGGC
	LutroCb_rev890	GAYAAGATTAGGGCCAATAC
	LutroCb_rev926	GAGGTGTGTAGCAGTGGGACG
