# Supplementary material for: Phylogeography of the smooth-coated otter (Lutrogale perspicillata): distinct evolutionary lineages and hybridization with the Asian small-clawed otter (Aonyx cinereus)
Source: Sci Rep. 2017 Jan 27;7:41611. doi: 10.1038/srep41611 (PMC5269716; doi:10.1038/srep41611)

Phylogeography of the smooth-coated otter (*Lutrogale perspicillata*): distinct evolutionary lineages and hybridization with the Asian small-clawed otter (*Aonyx cinereus*)

Beatrice Moretti, Omar F. Al-Sheikhly, Monica Guerrini, Meryl Theng, Brij K. Gupta, Mukhtar K. Haba, Waseem A. Khan, Aleem A. Khan, Filippo Barbanera

Supplementary Information

Table S1. Modern and archival sample size of this study. Data include country, locality/region for the origin of the sample in the wild, latitude/longitude (Lat. N/Long. E), number (*n*), sex (M, male; F, female), type of sample, year of collection and the captive institution or the museum where eventually the sample came from. Notes comprise additional information on each sample, when available. With the exception of blood droplets (Whatman®FTA®cards or EDTA), all samples were preserved in 96% ethanol and stored at - 40 °C at the Department of Biology of Pisa (Italy). MtDNA haplotypes are indicated for both 1140 (H) and 307 (h) bp-long Cyt-*b* sequences. GenBank accession codes are provided in the last column: when haplotypes H and h occur at the same time, the GenBank accession code refers to the longest sequence (H). Other: PTWRC, Phnom Tamao Zoological Park and Wildlife Rescue Center, Phnom Penh (Cambodia).

Country	Origin in the wild	Lat.N/Long.E	*n*	Sex	Sample type	Year	Zoo/Museum	Notes	Cyt-*b* haplotype		GenBank accession code
**Modern samples (*n* = 81)**											
Lutrogale perspicillata (*n* = 58)											
Bangladesh	Khulna Division	22°48'/89°14'	1	M	Spraint	2014	Dhaka Zoo, Bangladesh	-	H27	h13	LT593933
	Mohanpur, Rajshahi Division	24°33'/88°38'	1	M	Spraint	2014	Rajshahi Zoo, Bangladesh	-	H28	h14	LT593934
Cambodia	Unknown	-	8	-	Spraint	2014	Colchester Zoo, UK	Originally from PTWRC	H19, H24, H25	h5, h12	LT593923/27/28
	Unknown	-	3	2M, F	Spraint	2014	PTWRC, Cambodia	-	H19, H21	h5	LT593923/24
	Unknown	-	1	F	Spraint	2014	Wingham Wildlife Park, UK	Originally from PTWRC	H25	h12	LT593928
India	Nearby Surat, Gujarat	-	2	-	Spraint	2014	-	-	-	h16, h17	LT593911/12
	Tapti River, Surat, Gujarat	21°09'/72°45'	4	-	Spraint	2015	-	-	H29	h14	LT593935
	Nearby Patna, Bihar	-	2	-	Spraint	2015	-	-	H27	h13	LT593933
Iraq	Al-Baghdadiya Lake, Chebaeish, Central Marshes, Thi Qar Province	31°02'/47°03'	1	F	Skin	2008	-	From dead otter	H17	h18	LT593922
	Om Am Nyaj Lake, Al-Hawizeh Marsh, Maysan Province	31°37'/47°35'	1	M	Skin	2008	-	From dead otter	H17	h18	LT593922
	Abu Khasaf, Al-Hawizeh Marsh, Maysan Province	31°38'/47°38'	1	M	Skin	2012	-	From dead otter	H17	h18	LT593922
	Al-Hawizeh Marsh, Maysan Province	31°41'/47°36'	1	M	Skin	2014	-	From dead otter	H17	h18	LT593922
	Al-Hawizeh Marsh, Maysan Province	31°57'/47°68'	2	M, F	Skin	2014	-	From dead otter	H17	h18	LT593922
Laos	Nam Ngum, Vientiane	18°31'/102°32'	1	-	Hair	2014	Lao Zoo and Wildlife Sanctuary, Laos	From dead otter	H25	h12	LT593928

Malaysia	Peninsular Malaysia	-	1	F	Spraint	2014	Wingham Wildlife Park, UK	-	H26	h9	LT593932
Pakistan	Keti Shah Forest, Sukkur	27°48’/68°54'	1	-	Blood	2014	-	-	H27	h13	LT593933
	Nara Canal, Khairpur	26°27'/68°54'	1	-	Blood	2014	-	-	H27	h13	LT593933
	Jamrao Headwork, Nawab Shah	26°56'/68°58'	1	-	Hair	2014	-	-	H27	h13	LT593933
	Chotiari Dam, Sanghar	26°12'/68°59'	1	-	Hair	2014	-	-	H27	h13	LT593933
	Power House, Sanghar	26°24'/68°52'	1	-	Hair	2014	-	-	H27	h13	LT593933
	Machi Goth, Matiari	25°24'/69°20'	1	-	Hair	2014	-	-	H27	h13	LT593933
	Goth Baqir, Sanghar	25°52'/68°32'	1	-	Hair	2014	-	-	H27	h13	LT593933
	Nearby Badin	24°34'/68°50'	1	-	Hair	2014	-	-	H27	h13	LT593933
Singapore	Sungei Buloh Wetlands Reserve	1°26'/103°43'	3	-	Spraint	2014	-		H15	h21	LT593920
	Marina East, Marina Bay	1°16'/103°52'	3	-	Spraint	2014	-	-	H14, H15	h21	LT593919/20
	Punggol Reservoir	1°24'/103°53'	1	-	Spraint	2011	-	-	H15	h21	LT593920
	West Coast Park	1°17'/103°46'	1	M	Skin	2011	-	Road-killed otter (only sample)	H15	h21	LT593920
	Kranji Dam Reservoir	1°26'/103°44'	2	-	Skin; spraint	2015	-	Otter found dead in the wild (entire individual)	H15	h21	LT593920
	Off Stadium, Marina Bay	1°18'/103°52'	3	-	Spraint	2015	-	-	H15	h21	LT593920
	Serangoon Reservoir	1°23'/103°55'	5	-	Spraint	2015	-	-	H13, H15	h21	LT593918/20
Thailand	Bangkhuntien, Inner Gulf	13°34'/100°25'	1	M	Spraint	2014	-	-	H19	h5	LT593923
Vietnam	Unknown	-	1	M	Spraint	2014	Wingham Wildlife Park, UK	-	H23	h11	LT593926
Lutra lutra (*n* = 7)											
Iraq	“Abu Ajaj” Abu Al-Tayar Lake, Al-Hammar Marsh, Thi Qar Province	30°45'/47°01'	1	M	Skin	2008	-	From dead otter	H2	h1	LT593914
	Taq Taq, Erbil Province, Kurdistan	35°53'/44°35'	2	2M	Skin	2007	-	From dead otter	H1	h1	LT593913
	Taq Taq, Erbil Province, Kurdistan	35°53'/44°35'	1	-	Spraint	2014	-	From dead otter	H3	h1	LT593915
Italy	Policastro Bussentino	40°06'/15°32'	1	M	Tissue	2014	-	Road-killed otter (only sample)	H7	h2	LT593916
	Riserva Naturale di San Giuliano	40°37'/16°28'	1	M	Tissue	2014	-	Road-killed otter (only sample)	H7	h2	LT593916
	Vallo della Lucania	40°13'/15°15'	1	M	Tissue	2013	-	Road-killed otter (only sample)	H7	h2	LT593916
Aonyx cinereus (*n* = 16)											
Malaysia	Road from Krau to Jenderak Pahang, Pen. Malaysia	4°12'/101°58'	1	-	Hair	2006	National Museum of Natural History (Paris: MNHN TC-563)	Road-killed otter	H16	h22	LT593921
Unknown	-	-	4	-	Blood	2014	Basel Zoo, Switzerland	-	H11	h20	LT593917
Unknown	-	-	4	3M, F	Blood	2015	Perth Zoo, Australia	-	H11	h20	LT593917
Unknown	-	-	3	3F	Hair	2015	Edinburgh Zoo, UK	-	H11	h20	LT593917
Unknown	-	-	4	-	Spraint	2015	Ostrava Zoo, Czech Republic	-	H11	h20	LT593917

**Archival samples (n = 11)**											
Lutrogale perspicillata (n = 11)											
India	Kolkata, West Bengal	22°32'/88°25'	1	M	Skin	1955	Natural History Museum of Denmark (Copenhagen: CN 4712)	-	-	h15	LT593910
Indonesia	Medan, Sumatra	3°21'/98°24'	2	2M	Toe pad	1970	Natural History Museum (Vienna: NHM 66152, NHM 66153)	-	-	h10	LT593907
Laos	Thateng, Plateau des Bolovens, Balikhamxay Province	15°18'/106°18'	1	M	Skin fragment	1931	Field Museum of Natural History (Chicago: FMNH 38010)	-	-	h5	LT593902
	Pakse, Balikhamxay Province	15°06'/105°48'	1	F	Skin	1931	Field Museum of Natural History (Chicago: FMNH 38011)	-	-	h5	LT593902
Malaysia	Sandakan, Sabah, Borneo	5°45'/117°52'	1	F	Skin fragment	1887	Smithsonian Institution National Museum of Natural History (Washington D.C.: USNM 19173)	-	-	h9	LT593906
	Pulau Langkawi, Kedah, Pen. Malaysia	6°21'/99°43'	1	F	Skin	1899	Smithsonian Institution National Museum of Natural History (Washington D.C.: USNM 104437)	-	-	h8	LT593905
Nepal	Chisapani, West Nepal	28°37'/81°16'	1	M	Bone	1948	Smithsonian Institution National Museum of Natural History (Washington D.C.: USNM 290145)	-	-	h13	LT593909
Thailand	Unknown	-	1	-	Bone	1882	National Museum of Natural History (Paris: MNHN-ZM-MO 1882-2947)	-	-	h5	LT593902
	Bang Nara River, Mueang Narathiwat District	6°13'/102°02'	1	-	Skin	1933	Natural History Museum of Denmark (Copenhagen: CN 2531)	-	-	h9	LT593906
Vietnam	Mekong River, South of Ho Chi Minh	9°48'/106°03'	1	M	Skin	1924	Smithsonian Institution National Museum of Natural History (Washington D.C.: USNM 240483)	-	-	h12	LT593908

Table S2. Fisher global test for departure from Linkage Disequilibrium for each locus pair across all populations. No comparison was significant (Bonferroni correction: α = 0.05, α’ = α/45 = 0.0011).

**Loci pair**	χ^2	***df***	***P***
Lut435/Lut453	5.72	10	0.84
Lut435/Lut457	20.60	12	0.06
Lut453/Lut457	9.08	10	0.52
Lut435/Lut615	14.88	10	0.14
Lut453/Lut615	5.29	8	0.73
Lut457/Lut615	17.37	10	0.07
Lut435/Lut818	6.40	8	0.60
Lut453/Lut818	0.99	4	0.91
Lut457/Lut818	14.12	8	0.08
Lut615/Lut818	8.52	8	0.38
Lut435/Lut832	10.16	10	0.43
Lut453/Lut832	1.82	8	0.99
Lut457/Lut832	8.27	10	0.60
Lut615/Lut832	5.18	10	0.88
Lut818/Lut832	5.74	8	0.68
Lut435/Lut604	15.54	8	0.05
Lut453/Lut604	5.65	8	0.69
Lut457/Lut604	15.94	8	0.04
Lut615/Lut604	5.88	6	0.44
Lut818/Lut604	13.50	6	0.04
Lut832/Lut604	15.46	8	0.05
Lut435/Lut701	6.80	12	0.87
Lut453/Lut701	8.05	10	0.62
Lut457/Lut701	18.47	12	0.10
Lut615/Lut701	10.08	10	0.43
Lut818/Lut701	7.69	8	0.46
Lut832/Lut701	6.41	10	0.78
Lut604/Lut701	7.25	8	0.51
Lut435/OT19	3.95	10	0.95
Lut453/OT19	12.68	10	0.24
Lut457/OT19	2.68	10	0.99
Lut615/OT19	11.46	8	0.18
Lut818/OT19	4.69	6	0.58
Lut832/OT19	11.14	8	0.19
Lut604/OT19	4.51	8	0.81
Lut701/OT19	4.11	10	0.94
Lut435/OT17	13.93	6	0.03
Lut453/OT17	1.12	6	0.98
Lut457/OT17	11.55	6	0.07
Lut615/OT17	16.11	6	0.01
Lut818/OT17	10.26	6	0.11
Lut832/OT17	7.00	6	0.32
Lut604/OT17	11.65	6	0.07
Lut701/OT17	4.28	6	0.64
OT19/OT17	6.34	6	0.39

Table S3. Posterior Probability of membership for each Singapore otter to *L. perspicillata* (Q_I) and *A. cinereus* (Q_{II}) species as inferred by STRUCTURE (see Fig. 4b). Legend: *, the only individual assigned to *L. perspicillata* as parental species; **, otter found dead near Kranji Dam, Singapore (see also Table S1 and Fig. S1).

**Individual**	**Q_I** (*L. perspicillata*)	**Q_{II}** (*A. cinereus*)
Singapore 1*	0.94	0.06
Singapore 2	0.40	0.60
Singapore 3	0.70	0.30
Singapore 5	0.11	0.89
Singapore 6	0.61	0.39
Singapore 7	0.88	0.12
Singapore 8	0.39	0.61
Singapore 9**	0.21	0.79
Singapore 11	0.42	0.58
Singapore 12	0.11	0.89
Singapore 13	0.33	0.67
Singapore 14	0.08	0.92
Singapore 15	0.01	0.99
Singapore 16	0.77	0.23
Singapore 17	0.46	0.54
Singapore 18	0.35	0.65
Singapore 19	0.29	0.71
Singapore 20	0.44	0.56

Table S4. Additional Cyt-*b* sequences used in the alignments. MtDNA haplotypes are indicated for both 1131 (H) and 305 (h) bp-long sequence alignment: when haplotypes H and h occur at the same time, the GenBank code refers to the longest sequence (H). *, from Kurdistan (North Iraq), with nine unresolved nucleotide positions; **, sequence kindly provided by K.-P. Koepfli (Smithsonian Conservation Biology Institute, National Zoological Park, Washington, USA) and used in this study.

**Taxon**	**Country**	**Cyt-*b* haplotype**		**GenBank code**	**Literature record**
Aonyx capensis	-	H30	h23	AF057118	Koepfli & Wayne (1998)
Aonyx cinereus	-	H12	h20	AF057119	Koepfli & Wayne (1998)
Enhydra lutris	-	H31	h24	AF057120	Koepfli & Wayne (1998)
Hydrictis maculicollis	-	H32	h25	AF057125	Koepfli & Wayne (1998)
Lutra lutra	Poland	H3	h1	AB564050	Sato *et al*. (2012)
Lutra lutra	Norway	H4	h1	AF057124	Koepfli & Wayne (1998)
Lutra lutra	Korea	H9	h3	EF672696	Ki *et al*. (2010)
Lutra lutra	Iberian Pen.	H5	h1	EF689067	Fernandes *et al*. (2008)
Lutra lutra	Iberian Pen.	H6	h1	EF689068	Fernandes *et al*. (2008)
Lutra lutra	-	H8	h3	FJ236015	Jang *et al*. (2009)
Lutra lutra	-	H8	h3	NC011358	Jang *et al*. (2009)
Lutra lutra	-	H3	h1	X94923	Ledje & Arnason (1996)
Lutra sumatrana	-	H10	h4	EF472347	Koepfli *et al*. (2008b)
Lutrogale perspicillata	Iraq	H18	h19	JQ437613*	Omer *et al*. (2012)
Lutrogale perspicillata	Thailand	H20	h6	EF472348	Koepfli *et al*. (2008b)
Lutrogale perspicillata	Cambodia	H20	h6	EF472348	Koepfli *et al*. (2008b)
Lutrogale perspicillata	Cambodia	H22	h6	LT593925**	This study
Lutrogale perspicillata	Thailand	-	h6	LT593903**	This study
Lutrogale perspicillata	Thailand	-	h7	LT593904**	This study

Fernandes, C. A. *et al*. Species-specific mitochondrial DNA markers for identification of non-invasive samples from sympatric carnivores in the Iberian Peninsula. *Conserv. Genet.*, **9**, 681-690 (2008)

Jang, K. H., Ryu, S. H. & Hwang, U. W. Mitochondrial Genome of the Eurasian Otter *Lutra lutra* (Mammalia, Carnivora, Mustelidae). *Genes Genom.*, **31**, 19-27 (2009)

Ki, J. S., Hwang, D. S., Park, T. J., Han, S. H. & Lee, J.S. A comparative analysis of the complete mitochondrial genome of the Eurasian otter *Lutra lutra* (Carnivora; Mustelidae). *Mol. Biol. Rep.*, **37**, 1943-1955 (2010)

Koepfli, K.-P. & Wayne, R. K. Phylogenetic relationships of otters (Carnivora: Mustelidae) based on mitochondrial cytochrome b sequences. *J. Zool.*, **246**, 401-416 (1998)

Koepfli, K.-P. *et al*. Establishing the foundation for an applied molecular taxonomy of otters in Southeast Asia. *Conserv. Genet.*, **9**, 1589-1604 (2008b)

Ledje, C. & Arnason, U. Phylogenetic analyses of complete cytochrome *b* genes of the order carnivora with particular emphasis on the caniformia. *J. Mol. Evol.*, **42**, 135-144 (1996)

Omer, S. A. *et al*. Evidence for persistence and a major range extension of the smooth-coated otter, *Lutrogale perspicillata maxwelli* (Mustelidae, Carnivora) in Iraq. *Folia Zool.*, **61**, 172-176 (2012)

Sato, J. J. *et al*. Evolutionary and biogeographic history of weasel-like carnivorans (Musteloidea). *Mol. Phylogenet. Evol.*, **63**, 745-757 (2012)

Table S5. Proportional likelihoods and negative log-likelihoods values for the reconstruction of the ancestral areas for the *L. perspicillata* clade (node 44). Three constrained topologies at node 41 were tested (see Methods). Node numbers correspond to those of Supplementary Figure S1. *L.p.m*, *L. p. maxwelli*; *L.p.s.*, *L. p. sindica*; *L.p.p.*, *L. p. perspicillata*. Code regions: 0, Europe; 1, Middle East; 2, South Asia; 3, South East Asia; 4, Africa; 5, northern Pacific coast.

Constrained topology		0	1	2	3	4	5	Inferred ancestral area
(*L.p.s.*,(*L.p.m*, *L.p.p.*))	Proportional likelihoods	0.0415	0.1237	0.4423	0.3094	0.0415	0.0415	
(*L.p.s.*,(*L.p.m*, *L.p.p.*))	Negative log likelihoods	39.96	38.87	37.59	37.96	39.96	39.96	Equivocal
(*L.p.m.*,(*L.p.s*, *L.p.p.*))	Proportional likelihoods	0.0468	0.2097	0.1827	0.4669	0.0469	0.0468	
(*L.p.m.*,(*L.p.s*, *L.p.p.*))	Negative log likelihoods	39.16	37.66	37.79	36.86	39.16	39.16	Equivocal
(*L.p.p.*,(*L.p.m*, *L.p.s.*))	Proportional likelihoods	0.0447	0.2641	0.1412	0.4605	0.0447	0.0447	
(*L.p.p.*,(*L.p.m*, *L.p.s.*))	Negative log likelihoods	39.64	37.86	38.49	37.31	39.64	39.64	Equivocal

Figure S1. Historical biogeography of investigated taxa. The distribution of each haplotype (H1-H32, Supplementary Table S1) is given in the brackets and with colour boxes at the end of the branch. Pie charts at nodes show proportional probabilities that the common ancestor was distributed in a given area. The outcome for the very large majority of nodes (included node 44, *L. perspicillata* clade) was D = 100%. Other nodes: node 51 = 100% DE; node 61 = 75% D, 25% DE; node 62 = 87.5% DF, 12.5% DEF; node 63 = 100% DEF. Area code: A, Europe; B, Middle East; C, South Asia; D, Southeast Asia; E, Africa; F: northern Pacific coast. For the sake of clarity, the Posterior Probability (PP) values obtained in the majority rule consensus tree (created by SDIVA) with compatible groups with less than 50% support were reported for each node in the *L. perspicillata* clade. Politomy among the three *L. perspicillata* subspecies was unresolved as in Fig. 2 (prevailing topology: (*L. p. sindica*, (*L. p. maxwelli*, *L. p. perspicillata*.)) with PP = 0.34. See also Supplementary Table S5.

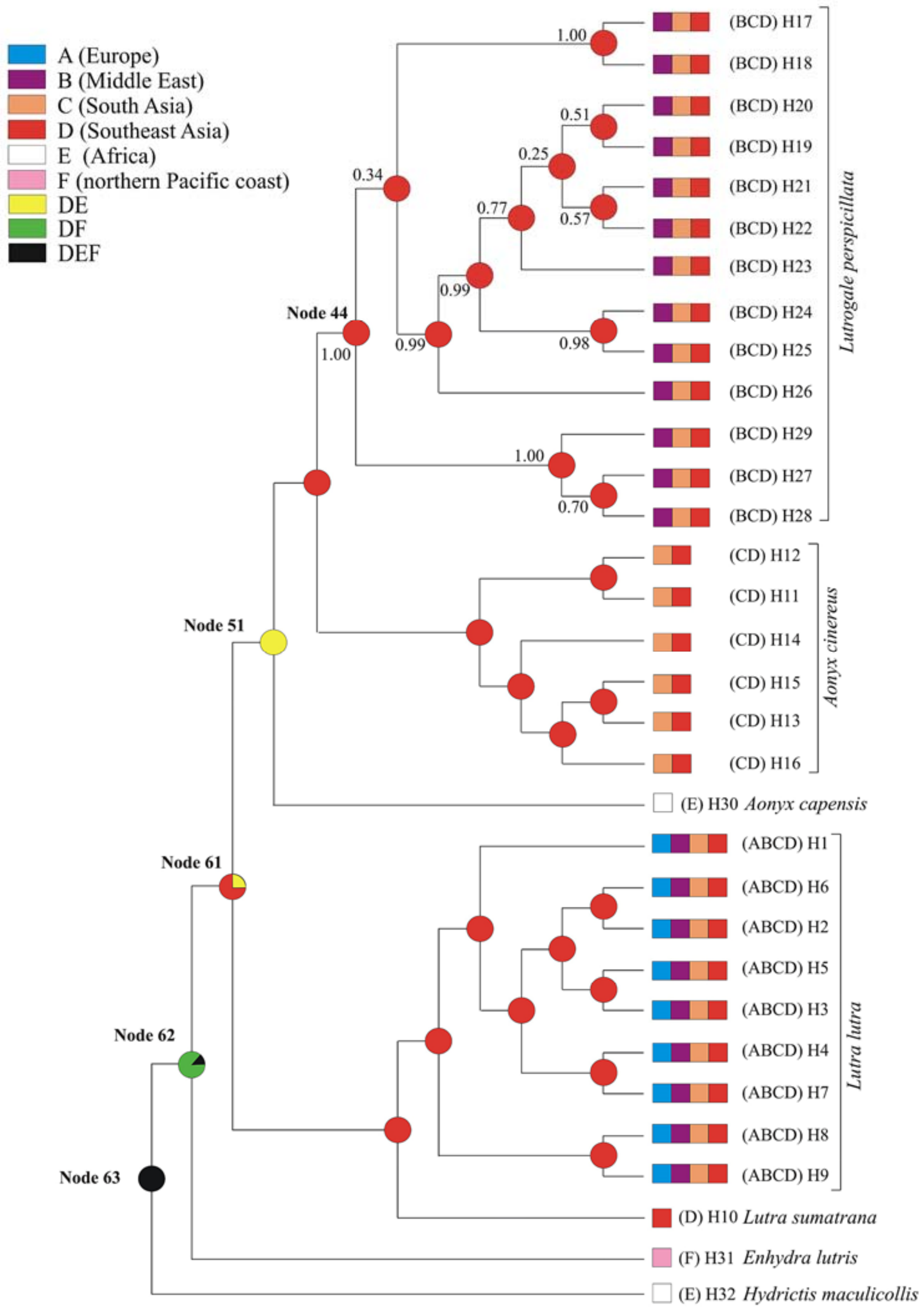

Supplement: Supplementary Information [file srep41611-s1.pdf]
